# Evolutionary pathway analysis and unified classification of East Asian lineage of *Mycobacterium tuberculosis*

**DOI:** 10.1038/s41598-017-10018-5

**Published:** 2017-08-23

**Authors:** Egor Shitikov, Sergey Kolchenko, Igor Mokrousov, Julia Bespyatykh, Dmitry Ischenko, Elena Ilina, Vadim Govorun

**Affiliations:** 1 Federal Research and Clinical Centre of Physical-Chemical Medicine, Moscow, Russian Federation; 2 Moscow Institute of Physics and Technology, Dolgoprudny, Russian Federation; 3 St. Petersburg Pasteur Institute, St. Petersburg, Russian Federation

## Abstract

Due to its rapid spread and association with the numerous outbreaks, the global spread of East Asian lineage of *Mycobacterium tuberculosis* strains presents a global concern. Although there were many attempts to describe its population structure, no consensus has been reached yet. To define unbiased classification that will facilitate future studies of this lineage, we analyzed the performance and congruence of eight different genotyping schemes based on phylogenetic analysis of 1,398 strains from 32 countries using whole-genome sequencing (WGS) data. We confirm that East Asian lineage comprises two major clades, designated proto-Beijing, which harbors unusual 43-signal spoligoprofile, and Beijing, with well-known spoligoprofile (deleted signals from 1 to 34). We show that different genotyping methods give high consistency results in description of ancient Beijing strains while the classification of modern Beijing strains is significantly divergent due to star-shaped phylogeny. Using WGS data we intersect different studies and for the first time provide balanced classification with well-defined major groups and their genetic markers. Our reconstructed phylogenetic tree can also be used for further analysis of epidemiologically important clusters and their ancestors as well as white spots of unclassified strains, which are prospective areas of research.

## Introduction

A nearly complete absence of horizontal gene transfer makes *Mycobacterium tuberculosis* (Mtb) population structure strictly clonal and hierarchical. Presently, it consists of seven phylogenetic lineages and usually smaller and more geographically delimited genetic families each of which is defined by unique event polymorphisms (SNP or deletions)^1^. Lineage 2 (or East Asian lineage) is arguably most widespread and the Beijing genotype family is its major component (13% of global *M. tuberculosis* population)^2^.

For the first time, the Beijing genotype was described in strains from the Beijing area in China which coined the name^3^. The strains were characterized by very similar IS6110-RFLP patterns and peculiar spoligotyping profile of only nine hybridization signals (35 to 43). Ten years later, a more inclusive definition of the Beijing genotype was suggested^4^; in particular, the spoligoprofile should have an absence of hybridization of signals from 1 to 34 and a presence of at least three of the spacers from 35 to 43 (according to the standard spoligotyping scheme). The Beijing genotype can also be identified based on phylogenetic analysis of the high-resolution 24 VNTR loci within the context of known reference strains (www.MIRU-VNTRplus.org).

The Beijing family was long believed to be a homogeneous group of strains, first of all based on the similarity of their IS6110-RFLP profiles (see representative examples in ref. 5), which in turn justified efforts to find more user-friendly and no less discriminatory molecular markers for these strains. The first evolutionarily meaningful subdivision of the Beijing genotype into large-scale phylogenetic lineages of ancient/atypical and modern/typical strains was proposed by Mokrousov in ref. 6. Further, in 2005, the same authors proposed a large-scale subdivision within Beijing genotype^7^ based on previously described insertion of IS*6110* in the NTF region^8^. The simplicity of analysis made the NTF-based approach widely used to discriminate between phylogenetic lineages of the Beijing genotype, although occasionally described discrepancies have cast a shadow of doubt over this marker^9^.

More recently, Tsolaki *et al*.^10^ and Gagneux *et al*.^11^ proposed a subdivision of Mtb into phylogenetic lineages mainly defined by large genomic deletions (regions of difference (RD)); the lineage 2 (defined by RD105) included the Beijing genotype (defined by RD207). Later, strains with RD105 but with unusual, complete 43-signal spoligoprofile^12^ were published and thus presented a minor part of the lineage 2 beyond the Beijing genotype proper. It should be mentioned that RD markers^10, 11^ did not distinguish between ancient and modern Beijing.

The advanced whole genome sequencing (WGS) technologies facilitated and accelerated global and local studies, and contributed to both gaining insight into high-resolution population structure and building the evolutionary scale framework of the Beijing genotype, in particular, and lineage 2 on a whole. Three kinds of studies should be noted: (i) based on polymorphisms in a (relatively) limited set of genes of the 3 R (i.e. Replication, Repair, Recombination) system^13, 14^; (ii) based on gene polymorphisms derived from comparison of the complete genomes^15^; and (iii) based on WGS data analysis^16–18^. Three mutator genes of the 3 R system (*mutT2*, *mutT4* and *ogt*) were used by Rad *et al*. to subdivide Beijing genotype into five groups and *mutT2* mutation in the modern Beijing group was supposedly associated with mutator phenotype^13^. The 3R-based approach was pursued on the expanded set of 56 such genes by Mestre *et al*.^14^ who subdivided Beijing strains into 26 groups; this was useful to follow the evolutionary pathway of this lineage but challenging in its interpretation since many subtypes included only single isolates. In its turn, SNP-based classification of Filliol *et al*.^15^ distinguished 11 types but some variable positions are presently regarded as uncertain and unsuitable^9^. Furthermore, the situation is complicated due to the fact that variable positions were given according to the earlier and what is worse, non-supervised version of the genome of reference strain H37Rv. Finally, typing schemes derived from WGS data suggest existence of 5^16^, 4^17^, and 8^18^ groups within lineage 2; here, a terminological discrepancy may be noted. Coll *et al*.^16^ species-wide study was based on WGS data and their subdivision was linked to the framework of the previously proposed phylogenetic lineages^10, 11^. On the other hand, Luo *et al*.^17^ focused on the East Asian lineage (lineage 2) and developed their classification and terminology based on independent analysis of the WGS data. A similar study was carried out by Merker *et al*.^18^ but they focused on the evolution of the Beijing genotype only and identified 3 and 5 subgroups within ancient and modern strains, respectively.

The major practical problem of such studies is that newly developed schemes partly or completely disregard previous knowledge and existing and established schemes. Consequently, a lack of any or adequate correlation between the old and the new studies makes it virtually impossible to assess the discriminatory capacity and evolutionary robustness of the particular typing methods. This latter issue is very important regarding public health. Phenotypic variation of strains from different genetic groups is a well-known and well-recognized fact and correct interpretation of typing results is a prerequisite for studies evaluating pathobiologically relevant properties such as drug resistance, virulence, transmissibility, mutator capacity etc. Additionally, there are open questions on specific polymorphisms involved in the formation of a subpopulation of the pathogen as well as validation and generalization of the existing techniques developed on the geographically delimited and partly biased datasets in new world regions.

In this study, we investigated *M*. *tuberculosis* lineage 2 classification methods and their performance and congruence using whole genome sequencing data of 1,398 strains. We correlated newly discovered and “old” molecular markers and superposed new schemes onto already long-used phylogenetic framework of the lineage 2 (~Beijing genotype). In practical terms, we aimed to facilitate communication between different research groups. We sought to clarify the evolutionary pathway of this epidemiologically and clinically significant lineage, and in particular, to highlight its known and as yet unknown epidemic clusters.

## Results

### Population structure and phylogenetic analysis

We performed the analysis of the evolutionary pathway of the Mtb lineage 2 on the 5,239 isolates from NCBI and ENA. Major phylogenetic lineages were determined based on SNP analysis^16, 19, 20^ (lin1 = 8.09%; lin2 = 29.18%; lin3 = 17.56%; lin4 = 43.42%; lin5 = 0.59%; lin6 = 0.67%; lin7 = 0.07%; unclassified = 0.49%). In total, we processed the whole-genome sequencing data for 1,398 lineage 2 strains from 32 countries and 13 independent studies and included them in our analysis (Table S1).

**Figure 1:**
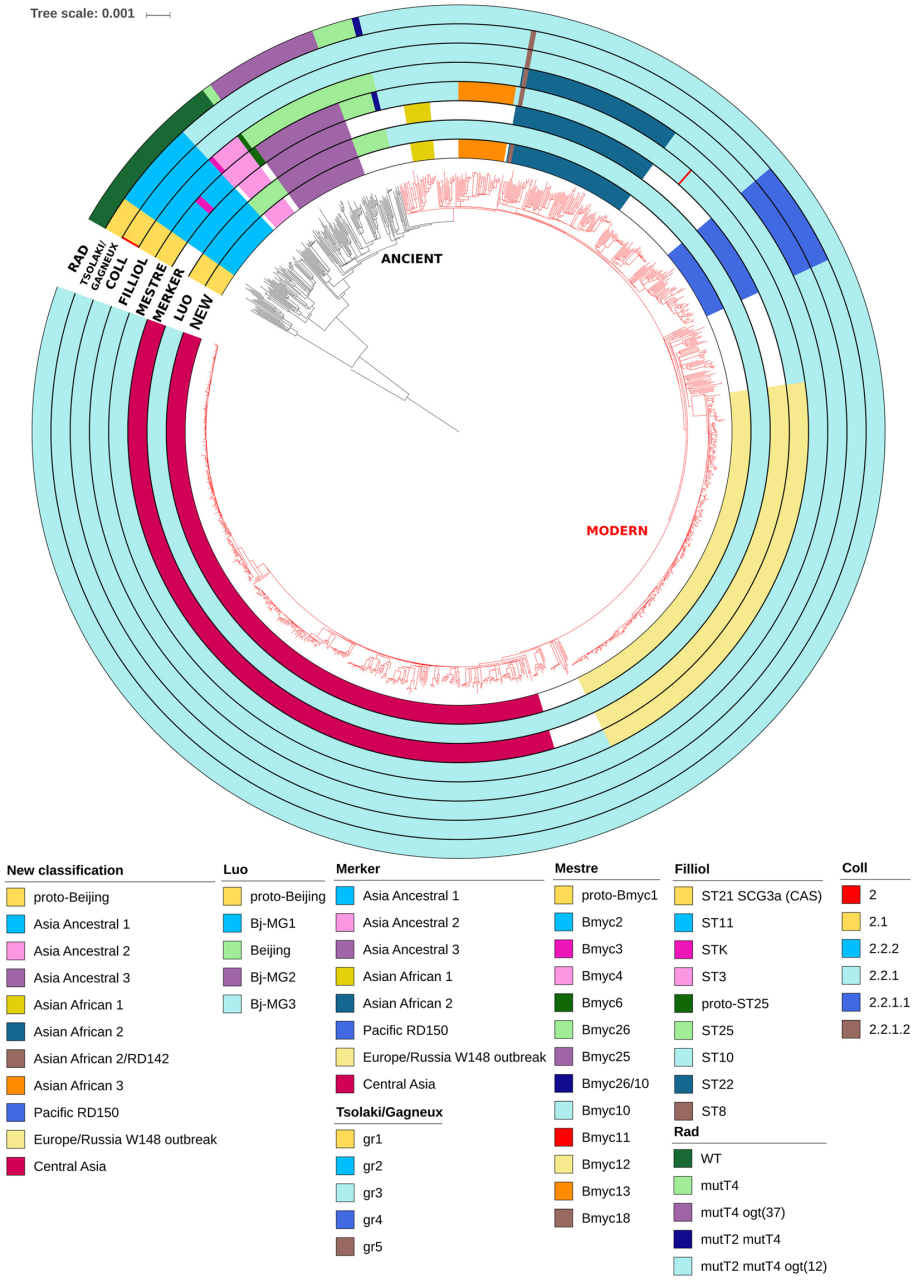
Phylogeny of 1,398 Mtb lineage 2 isolates. A total of 39,786 SNPs were used to reconstruct a maximum-likelihood phylogenetic tree. Colors in the outer circles indicate classification of the corresponding authors^7, 10, 11, 13–18^.

We identified 48,275 SNPs relative to the reference H37Rv strain. Strains of lineage 2 distinguish from others lineages by 117 specific SNPs, which is comparable to 106 and 124 SNPs from Coll *et al*.^16^ and Rose *et al*.^20^, respectively (Table S2). Overall, 1,601 SNPs were found per sample on average (range from 1165 to 1870), Overall, 1,601 SNPs were in average found per sample (range from 1165 to 1870), which corresponds to the SNP density of one SNP per 2.7 kb and consistent with previous estimations^21^. After excluding the repetitive, mobile elements, PE-PPE-PE_RGRS, drug-resistance associated genes and artifact SNPs linked to indels, we used remaining 39,786 SNPs to reconstruct a maximum-likelihood phylogeny of Mtb lineage 2 (Fig. 1).

### Genotyping methods comparison

In the current study, we focused on eight previously published genotyping methods/schemes^7, 10, 11, 13–18^. Six of them were based on SNPs analysis of different genome loci, while other two methods used regions of difference and insertion of IS*6110* in NTF region as genetic markers respectively (Table 1). We classified strains from our collection by every method and assigned eight unique identifiers (one for each classification scheme) to each strain (Table S1). The groups defined by particular methods and identified in the studied dataset, are shown in Table 1.

**Table 1:** Comparison of Typing Methods.

Reference	Genetic marker	Number of groups in this study/in reference study
Luo *et al*.^17^	SNPs	4/4
Merker *et al*.^18^	SNPs	8/8
Mestre *et al*.^14^	SNPs	11/26
Filliol *et al*.^15^	SNPs	6/11
Coll *et al*.^16^	SNPs	5/5
Tsolaki *et al*.^10^ */*Gagneux *et al*.^11^	RD	5/5
Rad *et al*.^13^	SNPs	4/5
Mokrousov *et al*.^7^	Specific insertion of IS*6110* in NTF region	2/2

On the next step, we depicted identified groups on the phylogenetic tree and obtained highly congruent phylogenetic relationships (Fig. 1). According to analysis, lineage 2 can be divided into two major phylogenetic clades represented by 20 and 1,378 samples, respectively. First clade was called lineage 2.1 (proto-Beijing clade) by Luo *et al*. According to Tsolaki *et al*.^10^/Gagneux *et al*.^11^ these strains belonged to group 1 and could be characterized by the RD105 deletion. In the meantime the majority of samples harbored a larger deletion (“extended RD105”), which affected genes Rv0068-Rv0075. Only one strain, used in Luo *et al*.^17^, was characterized by the typical deletion in this region. As for Mestre *et al*.^14^ scheme, we were able to identify an ancestral group relative to Bmyc1 which we labeled as proto-Bmyc1. At the same time, we could not find Bmyc1 among identified groups (Table S3). It is interesting, that strains from proto-Beijing clade belonged to ST21 SCG3a according to Filliol *et al*.^15^ (Table S4). According to *in silico* spoligotyping all lineage 2.1 strains carried an uncommon for Beijing strains spoligopattern, characterized by the presence of spacers 1 to 43 (Table S1).

The second major phylogenetic clade, which included the majority of samples (N = 1,378), was labeled lineage 2.2 according to Coll *et al*.^16^ and Luo *et al*.^17^ and represented the “classic” Beijing lineage, from which strains were characterized by the RD207 deletion. Earliest branch within the clade (50 samples) had intact *mutT2*, *mutT4* and *ogt* genes and was classified as a member of group 2, lineage 2.2.2, Bj-MG1 and Asia Ancestral 1 by Tsolaki *et al*.^10^/Gagneux *et al*.^11^, Coll *et al*.^16^, Luo *et al*.^17^ and Merker *et al*.^18^ studies respectively. As for Filliol *et al*.^15^, we assigned these strains to ST11. However, we were unable to distinguish ST11 from ST26, since we excluded position 909,166, located in the duplicated fragment of genome, from our analysis^22^. According to Mestre *et al*.^14^, strains of this branch could also be divided into Bmyc2 (45 samples) and Bmyc3 groups, with Bmyc2 as the ancestor of Bmyc3 (Table S3).

Further evolutionary step consists in acquiring the RD181 deletion and could be assigned to group 3 and lineage 2.2.1 by Tsolaki *et al*.^10^/Gagneux *et al*.^11^ and Coll *et al*.^16^, respectively. In the meantime, the transition from STK/ST3 to ST25 (by Filliol *et al*.^15^) and from Bmyc4 to Bmyc6 (by Mestre *et al*.^14^) was followed by acquiring a mutation in *mutT4* (codon 48), and, subsequently, Bmyc6 gave rise to Bmyc26 and Bmyc25. Asia Ancestral 2 group, identified in Merker *et al*.^18^, and Bmyc4 almost completely matched (Table S1).

The next major monophyletic group, which included 62 samples, characterized by mutations both in *mutT4* (codon 48) and *ogt* (codon 37), perfectly matched Bmyc25, Bj-MG2 and Asia Ancestral 3 identified by Mestre *et al*.^14^, Luo *et al*.^17^ and Merker *et al*.^18^, respectively. At the same time, Coll *et al*.^16^ and Tsolaki *et al*.^10^/Gagneux *et al*.^11^ did not discriminate this group. In addition, strains from this group, which harbored a mutation in *ogt* (37 codon), were assigned to ST25 from Filliol *et al*.^15^ classification. However, this group also included Beijing strains with intact *ogt* gene.

Finally, the largest monophyletic group (1,212 samples) in our collection, belonged to previously defined modern Beijing and harbored mutations in *mutT2* (codon 58), *mutT4* (codon 48) and *ogt* (codon 12), according to Rad *et al*.^13^ along with IS*6110* insertion in the NTF region, according to Mokrousov *et al*.^7^. Surprisingly, multiple insertion sites for IS*6110* were found in the NTF region even in some ancient Beijing strains hence this region may be defined as an IS*6110* hot spot (Text S1). In summary, modern Beijing included 8 separate groups - from 2^15^ to 5^18^ depending on the study and some of these groups were described in at least two studies (Fig. 1).

### Unified classification

To address the question of the goodness of each classification, we calculated HGDI, as a proxy of allelic diversity^23, 24^ (Table S5). Merker’s and Mestre’s classifications showed the highest, in relative terms, HGDI (0.77 and 0.56, respectively) and therefore we combined them to provide an unbiased unified classification of lineage 2, which we suggest to use for future studies (Fig. 1). The major drawback of these classifications was their inability to distinguish proto-Beijing strains, which severely restricted their applicability for lineage 2 classification. As we have mentioned already, we were not able to identify Bmyc1 (Mestre *et al*.^14^ scheme), although we found an ancestral group, which we labeled proto-Bmyc1. Equally important, Merker *et al*.^18^ included only Beijing strains carrying classical Beijing spoligotype in their study, which, by definition, excluded proto-Beijing strains.

Additional analysis of the two selected classification revealed their consistency between themselves and with other classifications. We found “well-defined” groups identified both in Merker *et al*.^18^ and Mestre *et al*.^14^ schemes, as well in other classifications (e.g. Asia Ancestral 3, which was also identified as Bj-MG2 or Bmyc25), and included such groups in our classification. Next, Asian African 1, Central Asia, Pacific RD150, Bmyc13 and Bmyc18 are also a part of our classification, since they represent clear-cut clusters with group-specific SNPs, which can be used for their identification. For the Bmyc13 group, we have studied countries of patients’ origin and found that this cluster represents Asia and Africa as well; thus we labeled it Asian African 3, following Merker’s naming. As for Bmyc18, even though this group comprises three samples and clustered within Asian African 2, we included it in our classification and labeled Asian African 2/RD142, since it harbors an RD142 deletion and has been described in four independent studies. However, we excluded Bmyc26 and Bmyc6 groups, since these groups represent an intermediate phase between ancient and modern Beijing, and we did not find any cluster-specific SNPs, which can be used for their detection. Thus we present a method for lineage 2 classification, which includes both proto-Beijing and Beijing clades, and has a higher discriminatory power (HGDI 0.79), comparing to other genotyping methods. Within Beijing clade we classify ten groups, where three of them belong to ancient Beijing group (Asia Ancestral 1, Asia Ancestral 2, Asia Ancestral 3) and seven belong to modern Beijing (Asian African 1, Asian African 2, Asian African 2/RD142, Asian African 3, Pacific RD150, Europe/Russia B0/W148 outbreak and Central Asia) (Fig. 1).

### SNPs intersection

**Figure 2:**
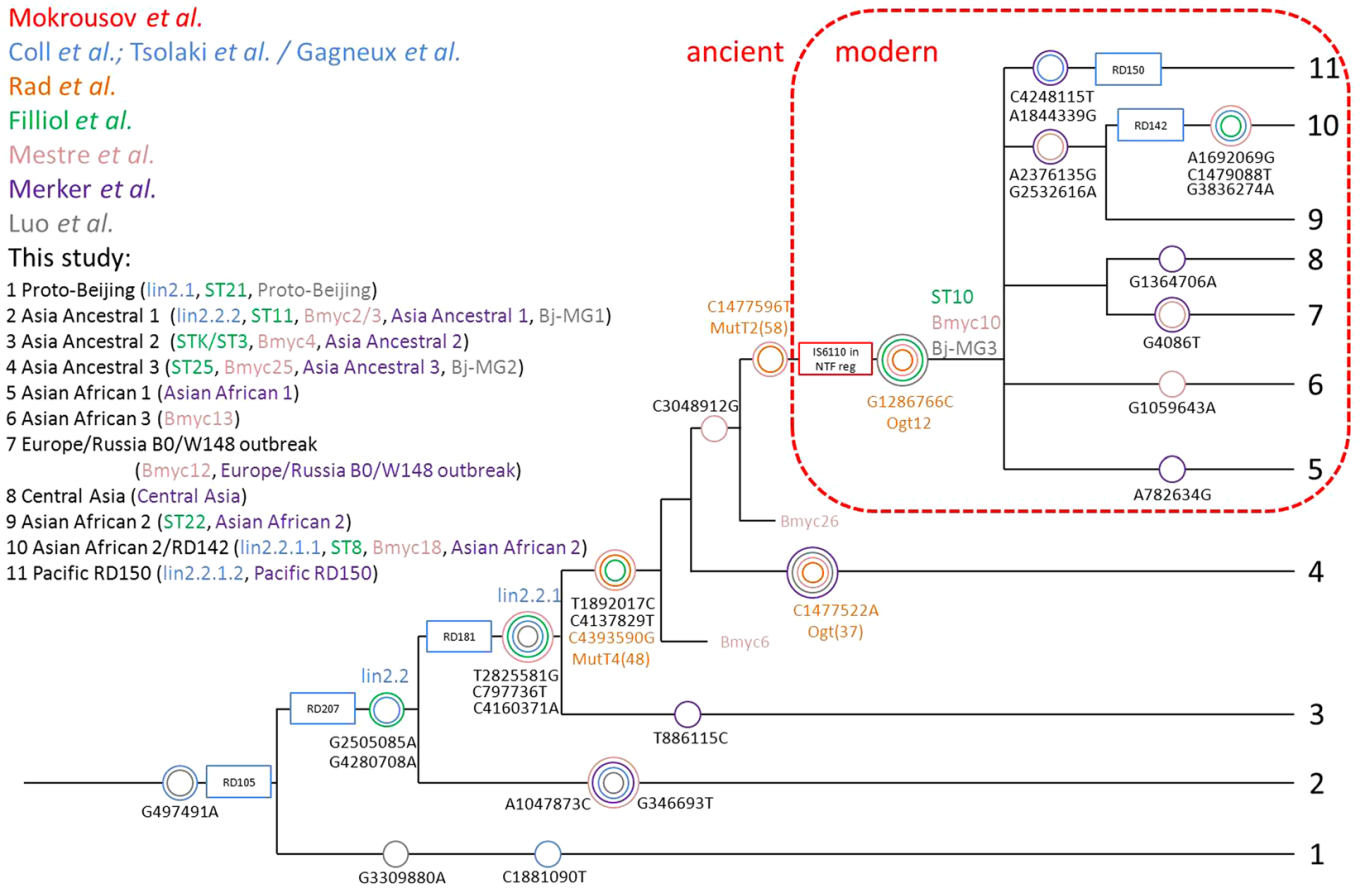
Mtb lineage 2 dendrogram representing the major phylogenetic groups and informative genetic markers.

During the analysis of the genotyping schemes and the phylogenetic tree we found that authors of the different genotyping systems tend to use the same SNPs for groups discrimination, but at the same time one group can be detected using different SNPs. For this reason, we summarized the specific SNPs from each of the studies, found intersections between them and suggest a set of markers to be used with our classification (Fig. 2, Figure S1, Table S6).

A mutation at pos. 497491(G > A) was used both in Coll *et al*.^16^ and Luo *et al*.^17^ studies for lineage 2 determination in general and is advisable to use for the first check of sample group. On a next step, two mutations can be used for detection proto-Beijing strains. Position 3309880 (G > A) (Luo *et al*.^17^) is preferable to pos. 1881090 (C > T) (Coll *et al*.^16^) when detecting proto-Beijing strains, since the latter allows to discriminate strains with the extended-RD105 deletion only.

Two mutations common for all Beijing strains were found and reported: pos. 2505085 (G > A) (Coll *et al*.^16^) and pos. 4280708 (G > A) (Filliol *et al*.^15^), and therefore can be used for Beijing strains detection. Asia Ancestral 1 group (Bj-MG 1, ST11) can be recognized using different mutations: pos. 1047873 (C > T), reported by Mestre *et al*.^14^, and pos. 346693 (G > T), reported by Luo *et al*.^17^, Coll *et al*.^16^ and Merker *et al*.^18^.

The following groups are characterized by the RD181 deletion and can be detected by numerous polymorphisms from different studies, although all of them are inherited by the derived/downstream sublineages. However, next group, Asia Ancestral 2, harbors a unique mutation at pos. 886115 (T > C), which can be used for detection. As mentioned above, Asia Ancestral 3 represents a well-defined group, which was identified by four different studies. However, all of them used the same mutation in *ogt* gene (codon 37, pos. 1477522 C > A).

As for the following groups, all of them belong to modern Beijing clade. This group carries numerous specific SNPs and can be recognized with mutations in *mutT2* (codon 58, pos. 1286766 G > C) and *ogt* genes (codon 12, 1477596 C > T). The remaining groups, defined within modern Beijing clade, harbor unique mutations and can be easily identified. Full list of markers, used in different studies and phylogenetic tree with major clades are shown in Fig. 2, Figure S1 and Table S6.

### Application to epidemiological studies

The increased availability of whole-genome sequencing resulted in its large-scale use for Mtb genotyping and partial replacement of traditional genotyping methods. These technologies allow studying the origin and the evolution of the pathogen, the accumulation of antibiotic resistance and disease outbreaks. For estimation of abundance of our collection and its applicability for further studies we analyzed seven tuberculosis outbreaks. They represented strains circulating in the former Soviet Union (FSU) countries (Clade B, Clade A and CAO clusters^18, 25^), Gran Canaria (strain GC1237^26^), South Korea (strain K^27^), and USA (strain 210 and HN878^28, 29^). In our study, we could detect and classify all the above mentioned clusters and outbreaks strains (Figure S2).

According to our analysis, Strain K belonging to Bmyc3 and all 5 samples from our collection, that were assigned to Bmyc3, were isolated from patients from South Korea (Table S1). GC1237 strain, included in Mestre *et al*.^14^, belongs to Bmyc25 (Bj-MG2, Asia Ancestral 3). However, from the phylogenetic analysis we identified several subgroups within Bmyc 25, and GC1237 was a member of only one of that clades. We assigned Strain 210 and HN878 strain to Bmyc18, Asian Africa 2, and ST8 (Mestre *et al*.^14^, Merker *et al*.^18^, and Filliol *et al*.^15^ schemes, respectively). As we have mentioned above, ST8 represents a subgroup within ST22, harboring the RD142 deletion. In total, we identified 105 samples from 12 countries from this group in our collection (Figure S2, Table S1).

Next, we analyzed strains from Clade B, Clade A and CAO. Collectively, these clusters were recently defined as East European sublineage of the Beijing family that is prevalent in Russia and other FSU countries^25^. Based on our analysis, we detected 15 sublineage-specific SNPs which differ these strains from other modern Beijing. Subsequently, East European sublineage is subdivided into 2 groups, one giving rise to Clade B (289 strains) and other giving rise to Clade A (177 strains) and CAO (79 strains) (Figure S2). These clusters show a restricted genetic diversity (Clade B mean pairwise distance of 28 SNPs, ±SD 10 SNPs, CAO mean pairwise distance of 22 SNPs, ±SD 6 SNPs, Clade A mean pairwise distance of 16 SNPs, ±SD 9 SNPs) and on average harbor 50–60 unique cluster-specific SNPs (Table S6), which contributes to the hypothesis of their recent formation. To check this hypothesis, and estimate the time of origin of the East European sublineage, we used GTR substitution model and a strict molecular clock prior of 1 × 10^−7^ substitutions per nucleotide per year and estimated that the most recent common ancestor of Clade B, Clade A and CAO arose around 180 years ago (95% highest posterior density (HPD) 140–210), which is also corroborated with recent estimation by Eldholm *et al*.^30^.

## Discussion

Due to the high prevalence, successfulness and the ease of detection, there have been many attempts to classify strains belonging to lineage 2 (East-Asian) and to clarify their evolutionary pathway. Studies of epidemic strains, aimed at examining transmission chains and identifying the factors contributing to their dissemination, are no less interesting. However, the major difficulties that a nonspecialist faces when goes through the tuberculosis literature is the fluctuating and evolving nomenclature concerning this lineage. In our study, we combined all of this data using our collection of 1,398 samples and presented the unified classification and evolutionary pathway of lineage 2 of Mtb.

At least 8 different genotyping schemes were proposed by leading scientific groups, based on different genomic markers, such as, IS6110-RFLP, RDs, SNPs datasets, VNTR and WGS studies^7, 10, 11, 13–18^ (Table 1). In our study, we confirmed that lineage 2 comprises 2 major clades, designated proto-Beijing, which harbors unusual spoligoprofile, and Beijing, with classic, well-known spoligoprofile. There is no consensus on dating the origin of this lineage and the opinions and hypotheses are contrasting. One such hypothesis is based on the assumption that the age of the human Mtb *sensu stricto* is 70,000 years^1^ and the age of lineage 2 is 30,000 years^17^, other estimates time to most recent common ancestor (TMRCA) of Beijing lineage as 6,000 years^18^. While citing these papers, many authors illustrate differences in TMRCA estimations. However, after detailed analysis, it is clear, that Merker *et al*.^18^ did not consider proto-Beijing strains in their study, so these two different estimations cannot be compared in a straightforward way. In its turn, Beijing clade is divided into ancient and modern groups, in which smaller groups can be clearly identified (Fig. 1).

Analysis of ancient Beijing strains population structure using different classifications revealed high consistency of different genotyping methods. We were able to identify clear-cut clusters, which represent groups identified by two or more independent studies (e.g. Asia Ancestral 1, Asia Ancestral 2, Asia Ancestral 3 according to our classification). Such groups harbor many unique SNPs and can be easily identified using any of them. On the other hand, we also found groups representing intermediate phases, for which we did not find cluster-specific SNPs. One of such groups is Bmyc26, which illustrates transition from the ancient to the modern Beijing. This transition is usually determined by mutations in *mutT2* (codon 58) and *ogt* (codon 12) genes. However, we also identified strains with mutation in *mutT2* and intact *ogt* gene and labeled these strains Bmyc26/10 as an intermediary group between Bmyc26 and Bmyc10 (Table S3). These strains were isolated from patients from China and were firstly described in Liu *et al*.^31^.

In its turn, the boundary between the ancient and the modern Beijing is less distinctly traced in the study of Coll *et al*.^16^ and Tsolaki *et al*.^10^/Gagneux *et al*.^11^ thus this makes their genotyping schemes less suitable for the differentiation of Beijing strains (Fig. 1). The key point is that both of these classifications are fully matched each other and their major drawback is that both ancient and modern strains of Beijing subtype can be labeled as Group 3/lineage 2.2.1. On the other hand, *in silico* genotyping based on IS*6110* in the NTF locus showed controversial results: multiple insertion sites of this element among ancient Beijing strains demonstrate this region to be IS*6110* insertion hotspot (Text S1). At the same time, we did not find any strains from ancient Beijing group that contained the IS*6110* insertion in a typical spot for modern Beijing, as it was described by Nakanishi *et al*.^9^. However, that study included more than 1000 samples belonging to ancient Beijing group in contrast to only 191 such strains in the present study. As for modern Beijing group, all samples belonging to it harbored IS*6110* in the left part of the NTF region and in general had less additional insertion elements in non-typical sites. These results suggested that particular IS*6110* insertion in the NTF region occurred independently in modern strains.

Phylogenetic relationships of modern Beijing strains are less clear compared to the ancient Beijing family. We noted a large number of contradictions and inconsistencies between different authors. Further analysis revealed that this situation was caused by star-shape phylogeny of the modern Beijing when new groups evolve independently. Consequently, during their attempts to clarify the evolutionary pathway, authors were only able to suggest different genotyping schemes, and variety of groups they received depended on the collection diversity. Therefore, in order to obtain a balanced classification, it is necessary to consider all classifications and carefully study identified groups, their unique SNPs and other genetic markers.

As in ancient Beijing group, individual groups were independently identified in several different studies (e.g. Pacific RD150 and Asian African 2). Pacific RD150 group is a part of the CC5 complex and is phylogeographically specific for the Pacific region^18^. Additionally we note that strains with the RD150 deletion are a clear-cut, distinct group. In this sense, our study differs from Faksri *et al*.^32^, who showed deletions of this region in other modern Beijing groups.

One more group is strains with the RD142 deletion. We identified only 3 such strains among our collection, but they clustered together with epidemic HN878 and strain 210 also harboring this deletion (Figure S2). In general, strains from this group clustered together as a part of a larger group, identified both by Filliol *et al*.^15^ and Merker *et al*.^18^ (ST22 and Asian African 2 respectively), but despite this, we included the Bmyc18 group (which we called Asian African 2/RD142) in our classification because of its importance in terms of epidemiology and a unique RD142 deletion.

Another epidemically important population is the East European sublineage of Beijing family^25^, widespread in the FSU countries and characterized by a high level of drug resistance. In this study, the origin of this sublineage was estimated to be 1847 CE (95% highest posterior density (HPD), 1809–1882 CE) and their closest relatives were within the phylogenetically basally located subgroup of modern Beijing strains. Subsequently, the sublineage is divided into two subpopulations. One of them gave rise to Clade B, which was initially designated B0^33^ and W148^5^ but also named СС2^18^, East European 2^17^ and ECDC0002^34^. Our estimation of the TMRCA of this lineage is 1960 CE (HPD 1944–1973 CE), which is similar to the previous estimation^30^. Meanwhile, due to the size of our collection, we were able not only to identify the above-mentioned clusters, but also to find and describe their closest ancestors, which is essential for understanding the reasons behind their epidemiological success. It is interesting to note that the vast majority of Clade B samples were from Russia and Belarus, with the closest related strains being isolated primarily from patients from China (Figure S2). This allows us to assume that the ancestral forms of Clade B were from China, which corroborates with the recent study of Yin *et al*.^35^.

The second branch of the East European sublineage, named the Central Asia group^18^ and East European 1^17^, was a more heterogeneous population than the first branch (mean pairwise distance of 80 SNPs, ± SD 36 SNPs), which is in consistence with previous studies^17, 18^. However, here we should also emphasize a number of features. This group is relatively older, compared to Clade B (1877 CE, HPD 1843–1901 CE) and, according to the Merker *et al*. study^18^, Central Asia group contained 31% of MDR strains, which also tended to cluster. According to our study, more than half of the Central Asia group strains were MDR (310 out of 506), with most of them also belonged to clustering samples of epidemic populations. For example, Clade A and CAO clusters contained 196 and 50 MDR strains from 224 and 80 strains of clusters, respectively. In its turn, the exclusion of samples belonging to these clusters from the Central Asia group results in decreasing the percentage of MDR strains to 31.7% (64 of 202), which corresponds to the average for lineage 2 excluding the East European sublineage (30% MDR samples, 149 of 505). The latter suggests that using any classification within a strictly delineated group, one can find more successful strains characterized by their geographical distribution (Clade A is one of the most common clusters in Russia, CAO cluster strains are most common in Central Asia), virulence and drug resistance.

## Conclusion

Our SNP-based phylogenetic analysis of a global collection of Mtb lineage 2 isolates suggests that the evolutionary pathway and branch development description, proposed by different groups, are consistent with regard to the ancient strains. At the same time, phylogeny analysis of the modern strains revealed great discrepancies and contradictions, which we examined in this study. Our results provide additional insights into phylogeny of Mtb lineage 2, since the whole-genome SNP tree revealed much more phylogenetic detail, such as strict relationship between groups, positioning of epidemiologically important groups and “blank spots” of noticeable clusters.

Our proposed classification allows identifying both proto-Beijing and Beijing strains. Beijing group is divided into two, ancient Beijing clade and modern Beijing clade, which consist of three (Asia Ancestral 1, Asia Ancestral 2, Asia Ancestral 3) and seven (Asian African 1, Asian African 2, Asian African 2/RD142, Asian African 3, Pacific RD150, Europe/Russia B0/W148 outbreak and Central Asia) (Figs 1 and 2) groups respectively.

We suggest that the classification proposed herein, as well as analysis of existing genotyping schemes evolutionary pathways should facilitate future studies of lineage 2 and will help to classify Mtb strain correctly. A more detailed study of the SNP tree together with phenotypic information might result in more accurate and robust clade assignment and may lead to better understanding of the molecular determinants and the selection forces that have contributed to the global success of the East Asian lineage.

## Materials and Methods

### Genome sequencing data

Whole-genome sequencing data of 5,715 Mtb isolates was obtained from National Center for Biotechnology Information (NCBI) and European Nucleotide Archive (ENA). The dataset consisted of thirteen independent WGS studies, available under accessions ERP000111^36^, ERP000124, ERP000192^25^, ERP000276^37^, ERP000436^38, 39^, ERP001731^1^, ERP002617^40^, ERP004677, ERP006989^18^, ERP013054^41^, SRA065095^42^, SRP051093^17^, and TB-ARC - Belarus.

In addition, a set of 5 samples, W-148 (CP012090.1), Strain K (CP007803.1), Strain 210 (ADAB00000000.1), GS1237 (ERR071082), and HN878 (NZ_CM001043), associated with tuberculosis outbreaks in different countries, was downloaded from NCBI. Genome H37Rv (NC_000962.3) was used as reference.

### SNPs calling

We aligned reads from whole-genome sequencing to the H37Rv (NC_000962.3) genome using Bowtie 2^43^. After, we sorted, indexed the aligned reads and converted them into a mpileup file using SAMtools^44^ and discarded samples with median read depth less than 30. We used VarScan^45^ to determine the variants in the remaining samples (n = 5,239) and MUMmer 3.20 with its nucmer and show-snps functions for the alignment of complete Mtb genomes to H37Rv^46^.

### Bioinformatics analysis

For further analysis, we made a custom R script. We assigned the Mtb samples to the main phylogenetic lineages on the basis of lineage-specific SNPs^16, 19, 20^. SNPs with a variant allele frequency of less than 90% or with coverage of less than 5 reads were discarded, as they are likely to originate from mapping errors. We annotated the remaining SNPs using the H37Rv annotation and classify them into synonymous and nonsynonymous. In addition, we filtered out SNPs in repetitive, mobile elements, PE-PPE-PE_RGRS genes and drug-resistance associated genes due to complexity of such regions^47^. From a group of samples which differ by less than 10 SNPs, we left only the one with higher mapping rate. For further validations we used Tablet^48^.

To create a maximum-likelihood phylogenetic tree with R phangorn package^49^ we concatenated remaining SNPs (n = 39,786) for each lineage 2 strain (n = 1,398) into an artificial sequence and used iTOL^50^ for visualization and annotation.

### BEAST evolutionary analysis

We selected 896 samples, representing Central Asia outbreak, CladeA, and CladeB, for computing dated phylogeny and divergence time using BEAST (v1.8.4)^51^. We used GTR model with gamma site heterogeneity model with 4 parameters and we defined lognormal prior distribution for the substitution rate (1 × 10^−7^, ranging from 9.3 × 10^−6^ to 1.7 × 10^−7^) thus allowing for ~0.5 SNP/genome/year^51^. We run chains of 10^7^ generations, sampled every 1000 was run and assessed convergence using Tracer, ensuring all relevant parameters reached an effective population size of >100.

### Spoligotyping, RD deletions, and IS*6110* analysis

Isolates assigned to lineage 2 (n = 1,398) were in silico spoligotyped from raw sequence files (fastq format) using SpoTyping software^52^. For the analysis of presence or absence of six LSP loci (RD105, extended-RD105, RD207, RD181, RD150, and RD142) we compared the mapping depth of the targeted region with the coverage of the corresponding flanking regions. If the average coverage of the targeted region was at least two fold lower than that of both flanking regions, a deletion was called, as described before^17^. We used ISMapper^53^ for the analysis of the IS*6110* insertion in NTF region and Integrative Genomics Viewer for visual control of the insertion position^54^.

## Electronic supplementary material

supplementary information

Table S1

Table S2

Table S3

Table S4

Table S6
