# Supplementary material for: Evolutionary pathway analysis and unified classification of East Asian lineage of Mycobacterium tuberculosis
Source: Sci Rep. 2017 Aug 23;7:9227. doi: 10.1038/s41598-017-10018-5 (PMC5569047; doi:10.1038/s41598-017-10018-5)

Evolutionary pathway analysis and unified classification of East Asian lineage of *Mycobacterium tuberculosis*

Egor Shitikov[1*], Sergey Kolchenko[1,2], Igor Mokrousov[3], Julia Bespyatykh[1], Dmitry Ischenko[2], Elena Ilina[1], Vadim Govorun[1]

1. Federal Research and Clinical Centre of Physical-Chemical Medicine, Moscow, Russian Federation.
2. Moscow Institute of Physics and Technology, Dolgoprudny, Russian Federation.
3. St. Petersburg Pasteur Institute, St. Petersburg, Russian Federation.

[*] corresponding author: egorshtkv@gmail.com

Text S1. Specific insertion of IS*6110* in NTF region

The 556 bp-long NTF region was firstly used for revealing of multidrug-resistant strain W of Mycobacterium tuberculosis prevalent in USA in 90s. The key feature of this region was its location between two codirectional IS6110 elements in W strains. It was proposed to detect these strains using multiplex PCR with appropriate primers[1]. However, in 2005, Mokrousov presented an elegant study[2], where it was defined that the absence of IS6110 insertion around the NTF region corresponds to the ancient Beijing type, whereas the presence of only IS6110 insertion at one end of the NTF region (determined by using MDR-6 primer) corresponds to the modern Beijing subtype. At the same time, this system is sensitive to the orientation of the primers, as well as PCR fragments of non-standard length can appear if insertion site is shifted.

During our analysis we noticed that this region contained not only insertion in the expected position but also multiple insertions in different sites around +/- 200 bp from the standard insertions site. In our case, 13 out of 186 ancient Beijing strains harbored an IS*6110* insertion in non-typical sites. We have observed that 3 insertions out of 13 were on the left of MDR-6 primer. Nevertheless it would have been impossible to get the amplification product and detect the insertion since an IS*6110* was in reversed orientation. As for modern Beijing group, all samples belonging to it harbored IS*6110* in the left part of the NTF region and in general had less additional insertion elements in non-typical sites. In addition, we were unable to detect any stain belonging to strain W type.

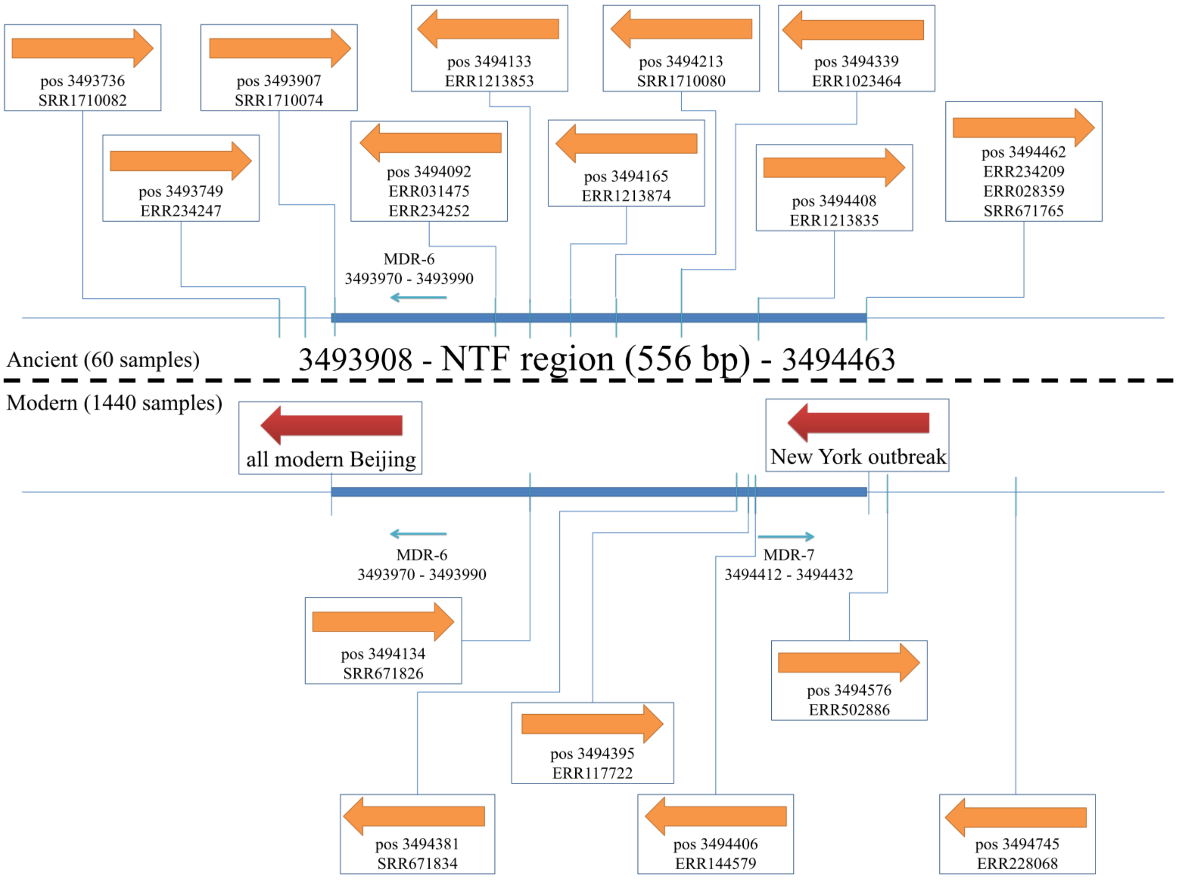

1. Plikaytis, B. B. *et al.* Multiplex PCR assay specific for the multidrug-resistant strain W of *Mycobacterium tuberculosis*. *J Clin Microbiol.* **32**, 1542-1546. (1994).

2. Mokrousov, I. *et al.* Origin and primary dispersal of the *Mycobacterium tuberculosis* Beijing genotype: clues from human phylogeography. *Genome Res.* **15**, 1357-1364. Epub 2005 Sep 1316. (2005).

Table S5. Hunter Gaston discriminatory index of studied and new classifications

Study	HGDI
Coll	0.18
Tsolaki	0.18
Filliol	0.36
Mestre	0.56
Luo	0.24
Mocrousov	0.23
Merker	0.77
Rad	0.24
This study	0.79

Figure S1. SNPs intersections according to different studies

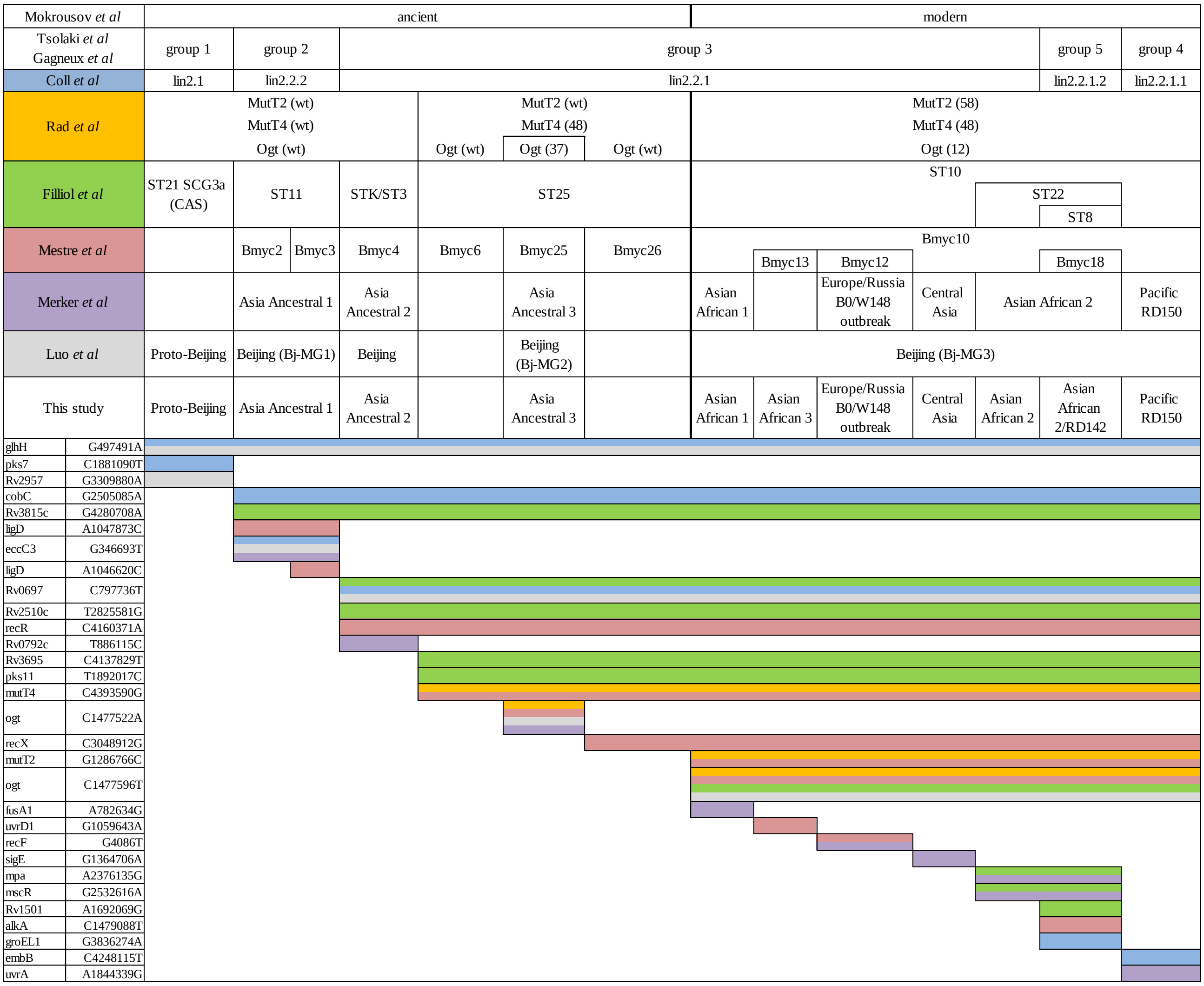

Figure S2. Phylogeny of 1,398 Mtb lineage 2 isolates with 5 epidemiological strains. Colors in the outer circles indicate the drug resistance pattern and country of origin of the corresponding strain. Three epidemiological clusters are labeled and highlighted as follows: red, Clade A; orange, CAO, violet, Clade B.

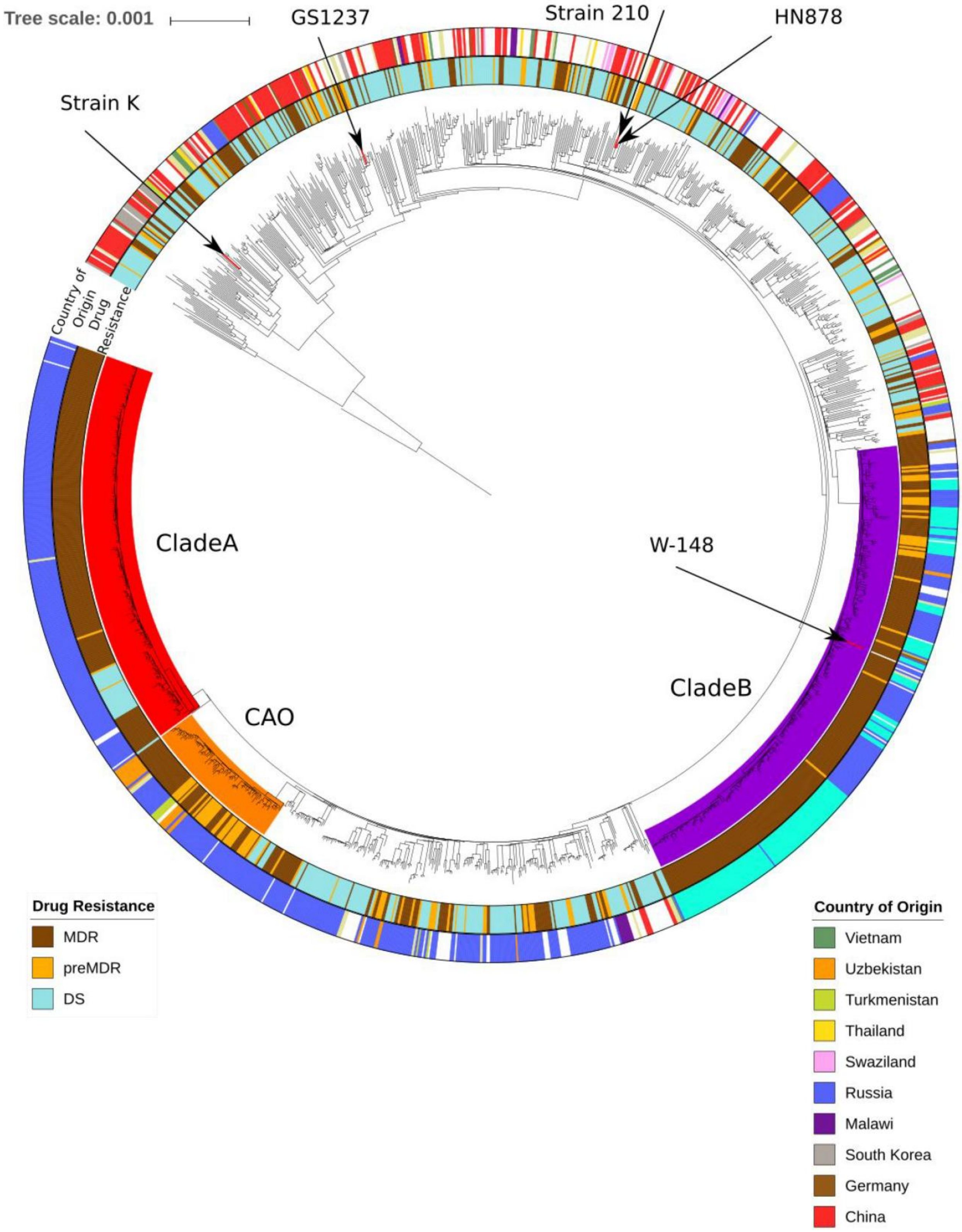

Supplement: Supplementary file 1 — supplementary information [file 41598_2017_10018_MOESM1_ESM.pdf]
